# Prevention of Scarlet Fever

**Published:** 1881-07-20

**Authors:** J. B. Garrison

**Affiliations:** Williamatte, Ark.


### PREVENTION OF SCARLET FEVER.

BY J. B. GARRISON, M. D., WILLIAMATTE, ARK.

More than two years ago I published the assertion that I firmly believed that the contagion of Scarlet Fever could be absolutely prevented. My statement at that time was based principally on my conception of the manner in which the transmission of the scarlet fever poison was effected; my method of prevention having been tested in a few cases only. Since that time, however, clinical experience has afforded me abundant opportunities of verifying my assertion that by a certain method of procedure in the treatment of scarlet fever patients, the contagious element of the disease is rendered innocuous. For proofs of the instances I shall hereafter detail, I refer to Dr. E. Visart, of DeWitt, Ark., together with the residents of that town and vicinity.

In the fall of 1878, Prairie township, adjoining the one in which I lived, was visited with an epidemic of scarlet fever, attended with considerable fatality. I proclaimed publicly that I would prevent its spread in that locality to which my practice was confined.

The first case to which I was called was a daughter of A. B. Beeler, a druggest residing in DeWitt. . She had been visiting with her mother in the infected district, was brought home and survived a severe attack of scarlet: ever of the anginose variety. Five persons in this family were constantly exposed to actual contact with this patient. Two of them slept in the same room, and part of the time in the same bed with her, and, although they had not previously had scarlet fever, none of them contracted it. No quarantine was enforced, and several unprotected children visited the patient during her illness, and no case occurred in consequence of the exposure. Soon after I was called to see five patients who had scarlet fever in the family of Mr. John Watkins, living in the country three miles from DeWitt. They had been on a visit to some relatives who were afterwards found to have scarlet fever. The disease ran its usual course, even to the desquamation of the thick cuticular covering of the palms of the hands and soles of the feet. One of the patients, an infant, was a corpse in the house at my first visit. Although there were seven other members of the family who had not previously had the fever, who lived constantly, ate and slept in the only two rooms of the house in which the fever patients were confined, nursed them and slept in the same beds with them, yet no other case occurred in that family.

These are but two instances selected from more than twenty, occurring in five epidemics (or which would have been) in which I have given the method a fair trial. I cite these two from the fact that I called attention to them publicly at the time of their occurrence, (so as to have the means of verification) and also publicly announced my ability to arrest the spread of the disease in the community.

I declared more than ten years ago my belief that almost the sole means by which scarlet fever was propagated was by direct inoculation of desquamated cuticle from the scarlet fever patient to the exposed individual. Experience has confirmed me in this belief.

Hence, my practice is to destroy the poison contained in and render inert, the light, furfuraceous desquamated cuticle by disinfecants.

I do this by annointing the entire body of the patient with carbolized lard or cosmoline.

| | | |
|---|---|---|
| ℞ | Adipis, aut olei petrolei, | 2 oz. |
| | Acid carbolic pur | 1 drachm. |

Sig. Annoint thoroughly the entire body of the patient twice in 24 hours.

For further precaution I also direct a carbolated gargle :

| | | |
|---|---|---|
| ℞ | Potass chlor. pulv | 2 drachms. |
| | Acid carbolic | 2 " |
| | Glycerinæ | 2 oz. |
| | Aqua camphoræ | 6 oz. |

Sig. Use as a gargle every 3 or 4 hours, oftener if indicated.

I also direct the exhibition of carbolic acid, *per orem*, in the average dose of two grains every four hours, for an adult. The atmosphere of the room, under these conditions, being loaded with carbolic vapor from the inunctions and gargles, precludes the necessity of the inhalation of a carbolic solution.

Patients generally express relief from the inunctions and gargles, nor does the prophylaxis interfere with any other indications for treatment.

I have no stereotyped treatment for the disease. I treat the individual patient with veratrum, digitalis, ice, cold water, or brandy, opium and sinapisms, as may be indicated.

Nevertheless I opine there are few cases of scarlatina in which chlorate of potash and quinine will not prove beneficial.

Several years ago I communicated my views and experience on this subject to the greatest of all therapeutists and best of medical teachers as well as of men, (in my estimation) Prof. Samuel G. Armor, of Long Island College Hospital, Brooklyn, N. Y., who did me the honor to say that he was teaching my method of prevention to his class.

Quite a number of physicians to whom I have personally explained my plan of prophylaxis, report results equal to my own.

A year or two since I saw an article from some eastern professor recommending lard alone, used by inunction, as preventive of the spread of scarlet fever. This is doubtless true to a certain extent, as it would agglutinate the fine squamæ which might otherwise be carried to *nidi* favorable to the deposition of their poisonous burden. It would not, however, answer in cases of actual contact.

More recently Prof. Jules Simon, in a lecture delivered at the Hospital des Enfants Malades, Paris, declares that "scarlet fever is contagious principally through the pellicules of epidermis which become detached." (See Medical and Surgical Reporter, Philadelphia, March 26, 1881.) This corroborates my theory, and I am confident that a trial is all that is needed to convince any physician of the certainty of this method of prophylaxis against the contagion of scarlatina.

I may further state, in this connection, that, in 1872, I treated a case of variola, using carbolized inunctions; and, it being an isolated case, in the country, many persons visited it without knowing the nature of the disease. All unvaccinated individuals who visited the patient prior to the use of the inunctions, contracted small-pox; those exposed subsequently escaped.—*Indiana Medical Reporter.*

